# Efficacy and cost-effectiveness of intradiscal methylene blue injection for chronic discogenic low back pain: study protocol for a randomized controlled trial

**DOI:** 10.1186/s13063-015-1058-6

**Published:** 2015-11-21

**Authors:** José W. Geurts, Jan-Willem Kallewaard, Alfons Kessels, Paul C. Willems, Henk van Santbrink, Carmen Dirksen, Maarten van Kleef

**Affiliations:** Department of Anaesthesiology, and Pain Medicine, Maastricht University Medical Centre, Mailbox 5800, 6202 AZ Maastricht, The Netherlands; Rijnstate Hospital, Department of Anaesthesiology and Pain Management, Mailbox 9555, 6800 TA Arnhem, The Netherlands; Department of Clinical Epidemiology and Medical Technology, Maastricht University Medical Centre, P. Debeijlaan 25, 6229 HX Maastricht, The Netherlands; Department of Orthopaedic Surgery, Maastricht University Medical Centre, Mailbox 5800, 6202 AZ Maastricht, The Netherlands; Department of Neurosurgery, Maastricht University Medical Centre, Mailbox 5800, 6202 AZ Maastricht, The Netherlands; ‘Department of Neurosurgery, Atrium Medisch Centrum Heerlen’, Heerlen, The Netherlands

**Keywords:** Intervertebral disc, low back pain, methylene blue, outcome assessment (health care), pain, Treatment Outcome

## Abstract

**Background:**

Low back pain (LBP) is a common health problem and a substantial part of LBP is presumed to be attributable to degeneration of the intervertebral disc. For patients suffering from intractable discogenic LBP, there are few evidence-based effective interventional treatment options available. In 2010, the results of a randomized controlled trial (RCT) were published concerning “intradiscal methylene blue injection” (IMBI), in which this intervention appeared to be very successful in relieving discogenic pain. Therefore, we decided to repeat this study to investigate whether we could replicate the published results. The results of our preliminary feasibility study gave reason to set up an RCT. The aim of this RCT is to evaluate if IMBI is a more effective treatment of discogenic low back pain as an intradiscal placebo intervention, and furthermore, to assess the cost-effectiveness of this intervention.

**Methods/Design:**

Consecutive discogenic low back pain patients referred to four specialized pain treatment facilities are being screened for eligibility. After a positive standardized provocation discography and informed consent, patients are randomized into two groups. The treatment group receives an intradiscal injection with methylene blue, lidocaine, and contrast, and the control group receives intradiscal isotonic saline with lidocaine and contrast.

Main outcome measures are pain at the 6-month follow-up, patient’s global impression of change, cost-effectiveness, quality of life, disability, and analgesic intake.

**Discussion:**

The importance of this study is emphasized by the fact that for intractable discogenic low back pain patients, evidence-based effective pain treatments are rare. If this study establishes clinical success and cost-effectiveness, IMBI could become the “pain treatment of choice” for a selected group of patients with chronic discogenic low back pain for whom noninvasive treatment options have failed.

**Trial registration:**

National Trial register NTR2547

Registered at 29 September 2010 and 31 March 2014.

## Background

Low back pain (LBP) is a common health problem; more than 70 % of the population in industrialized countries will experience low back pain at least once in their life [1]. In most patients, LBP is assumed to resolve spontaneously, although in a recent systematic review it was shown that 60 % of patients who went to the general practitioner for their recent onset LBP, still suffer from back pain one year later [2]. LBP diminishes the quality of life of the individual patient substantially [3]. Furthermore, low back pain is also an economical problem for it imposes a substantial financial burden on society [4–7]. A substantial part of LBP is presumed to be attributable to degeneration of the intervertebral disc [8–10]. In a normal disc, nerve fibers are present only in the outer layers of the annulus fibrosis [11]. In contrast, in a degenerated disc, nociceptive nerves have been shown to grow into the inner layers of the annulus fibrosis along degenerative fissures [12–14]. Stimulation of these nerve endings with inflammatory mediators is assumed to produce pain [13, 15]. A positive provocative discography along with morphologic signs of disc degeneration, that is, annular tear grade II to IV according to the modified Dallas Classification, as seen during the discography procedure, is assumed to confirm the diagnosis of discogenic pain [16].

The treatment of discogenic chronic low back pain remains controversial. Patients who do not respond to conservative therapy may undergo various treatments, varying from interventional chronic pain treatment to fusion surgery, generally with variable and unpredictable results [17].

In 2010, results of a randomized placebo-controlled trial concerning intradiscal methylene blue injection (IMBI) were published [18]. It showed a clinically meaningful pain reduction in 89 % of IMBI-treated patients.

The rationale for this treatment is that methylene blue (MB) is neurolytic. Intradermal MB injection has shown by electron microscopy to be able to destroy dermal nerve endings [19]. By injecting methylene blue into the nucleus pulposus, it spreads into radial fissures where it can destroy the nerve endings or nociceptors that have grown into the painful disc. The authors and commentating author concluded that this treatment was promising but that it is imperative that more high-quality studies are performed to determine whether the results of this study can be reproduced.

In 2011, the Departments of Anesthesiology and Pain Medicine of Maastricht and Arnhem in the Netherlands started a pilot study to acquire information about effect size, recruitment strategies, acceptability of intervention, study burden, and safety of IMBI. The pilot indicates that IMBI could be effective (40 % responded to the treatment) in a well-selected group of patients suffering discogenic pain.

The proposed placebo controlled randomized clinical trial (RCT) was designed to investigate whether the results of the aforementioned RCT on IMBI can be replicated and additionally, to assess the cost effectiveness of this intervention.

## Methods/Design

A double-blind placebo controlled randomized clinical trial (RCT) will be conducted in four interventional pain centers in the Netherlands by certified pain specialists. The RCT is approved by European Union Drug Regulating Authorities Clinical Trials (EudraCT) and the medical ethics committee (METC) of the Maastricht University Medical Centre; registration number NL325 11.068.10 and 10-2-055, respectively. Informed consent will be obtained from all participants.

This study is sponsored by ‘The Netherlands Organisation for Health Research and Development’ (ZonMw), registration number 836011026.

Clinical trials registration number is NTR2547.

### Study population

Consecutive patients with chronic lumbar axial pain referred to the specialized pain centers are assessed for eligibility. Eligibility criteria are a history consistent with lumbar discogenic pain (for example, predominant axial pain produced on lumbar motion, significant functional limitation in sitting duration and tolerance); low back pain for at least 6 months, and in at least the last 6 weeks a poor response to treatment that is, analgesics, physiotherapy, facet blockade and radiofrequency therapy; age between 18 and 66 years; neurological exam without motor deficit; pain intensity of at least 5 measured with the Numeric Rating Scale (NRS) 1 to 10 [20].

Exclusion criteria are discogenic pain confirmed on more than two levels; extruded or sequestered herniated nucleus pulposus; previous lumbar surgery or invasive intradiscal procedures on suspected levels; symptomatic lumbar spinal stenosis; grade 1-2 spondylolisthesis. Further exclusions include BMI (Body Mass Index (kg/m^2^)) of ≥ 35, pregnancy, coagulopathy or oral anticoagulant therapy (except low-dose acetylsalicylic acid) in conditions that do not allow for a temporary discontinuation, and infection. Patients who are incapable of following verbal or written instructions or with psychiatric problems potentially interfering with cooperation in the study will be excluded as well.

Magnetic Resonance Imaging (MRI) must have been performed in the last 12 months before inclusion to rule out severe disc degeneration as evidenced by a > 50 % disc height loss at the affected level [21]. Patients undergo a provocative discography to confirm or rule out discogenic origin of the lumber pain [22–24]. The provocative discography will be conducted according to ISIS/IASP guidelines and with the use of a pressure- and velocity-controlled disc stimulation device (CDS) (Neurotherm™, Wilmington DE, US) [23]. In provocative discography, annular tears according to the Modified Dallas classification of annular tears scheme grade 1 to 4 (see Table 1) in either one, or at most two, discs must be present [13, 21]. Concordant pain provocation must be present in the disc(s) at pressures of less than 50 PSI above opening pressure, of at least NRS 7 (of 10) or ≥ 70 % reproduction of worst spontaneous pain [25]. At least one adjacent lumbar disc should perform as a negative control disc.

**Table 1:** Modified Dallas Classification [16]

Grade	Grade Description
0	Contrast is within the nucleus
1	Extravasation in the inner 1/3 of the annulus
2	Extravasation in the middle 1/3 of the annulus
3	Extravasation in the outer 1/3 of the annulus
4	More than 30 degrees of annular circumference
5	Extra-annular contrast

Patients who fulfil the eligibility criteria for this study are asked to participate in the study.

### Intradiscal methylene blue injection (IMBI) versus placebo

**Fig. 1:**
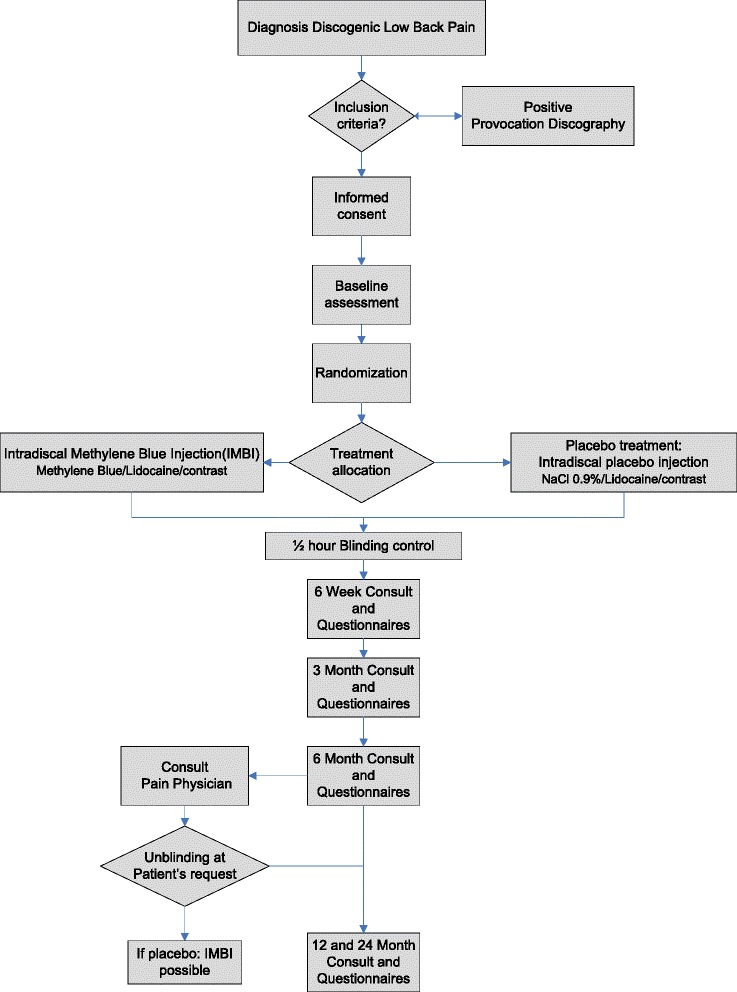
Trial design and patient flow

After written informed consent, patients will be randomized 1:1 for the treatment or control group Fig. 1. The treatment will be performed in the daycare surgery facilities of the participating specialized pain centers. To standardize this procedure and to ensure equal quality of treatment application, all participating physicians are trained in performing the IMBI intervention according to protocol.

Intradiscal methylene blue injection (IMBI) treatment consists of intradiscal injection with 1 ml of methylene blue, 0.5 ml of lidocaine hydrochloride 2 %, and 0.5 ml contrast dye (Iohexol-Omnipaque 300). The placebo treatment consists of 1 ml isotonic saline, 0.5 ml contrast dye, and 0.5 ml of lidocaine hydrochloride 2 %. This solution will be injected in the affected disc(s) under control of pressure (maximum of 100 PSI), velocity (0.02 ml/s), and volume using a CDS. During this procedure, the patient remains blind for the study-fluid. After the treatment, the patient has bed rest for at least 2 hours and during that time will be held in observation at the daycare surgery unit. Data will be collected at 6 weeks and at 3, 6, 12, and 24 months after the intervention, and patients will be seen by a physician or research nurse at each time-point to monitor patients’ adherence to the protocol.

### Allocation concealment

Both patients and post-intervention observers, such as pain managing nurses and physicians, are to remain blinded to the treatment or control intervention for at least 6 months. Therefore, during the intervention the interventional pain specialist takes care that the patient does not see the injected fluid. By asking the patient half an hour after the procedure what medication they think they received - either 1) methylene blue, 2) placebo or, 3) “I really don’t know” - the efficacy of the blinding procedure shall be checked. Furthermore, the same question will be repeated in the online patient questionnaire at 6, 12, and 24 weeks. Six-months post-intervention, at the patient’s request, patients from the control group who still suffer pain can receive the IMBI treatment.

### Objectives

Patients are assessed at baseline, at 6 weeks, 3 months, and at the primary end-point of 6 months, and furthermore, at the follow-up of 12, and 24 months.

Primary outcome measures is average pain at 6 months, measured by a pain diary with numeric rating scales (NRS) three times a day for 4 consecutive days. Success of the intervention is defined as pain reduction of at least 30 % at 6 months.

Other study variables include the Patients Global Impression of Change (PGIC) measured by a 7-point Likert Scale and number of adverse and serious adverse events [26]. Societal cost consisting of hospital cost and costs outside the hospital are measured by means of cost questionnaires. Disability is measured by the Oswestry Disability Index [26], Quality of life (QOL) by the Rand-36 and EQ-5D [26]. Information about pain characteristics is collected using the McGill Pain Questionnaire [26], and information about analgesics use with a diary at each time-point for 4 days. The potential predictive value, for success or failure of the intervention, will be explored with use of baseline variables, MRI classification, and registered variables of the provocative discography [26, 27].

With the exception of the pain and medication diary, and the health-related quality of life EuroQol (EQ-5D) questionnaire, which are presented to the patient in a booklet, all data will be collected by web-based questionnaires software, SelectSurvey. (NETv4.075.011© Copyright 2008 ClassApps.com) and MACRO (version 4.1.2.3750© 1999-2012 InferMed Limited, London, UK).

### Randomization and stratification

The randomization of study patients in either the treatment or the control group will be done with software for randomization of clinical trials ALEA (version 2.2 CTCM/ALEA). The stratification factors, that are used in the randomization procedure to assure that potential confounders are equally distributed between the two randomized groups, are study center; gender; dichotomized disc levels (1) L3/L4 to L4/L5 and (2) L5/S1; and dichotomized Modified Dallas criteria (1) 1 to 2 and (2) 3 to 4. The software automatically sends an e-mail with the randomization results to the un-blinded physician who will perform the IMBI treatment.

### Sample size

The sample size of the RCT is determined on the basis of success rates in the previous RCT [18] and our pilot [28]. We assume that 50 % of patients in the treatment group will be successfully treated. Success is defined as 30 % pain reduction at the 6-month follow-up. In the control group, we assume a 20 % success rate.

Given the invasive character of the IMBI procedure, we consider that the treatment group should be at least 30 % more successful than the control group to reach minimally clinical relevance. Furthermore, we assume 10 % drop out per group.

For a power of 80 % and a level of statistical significance of 5 %, the minimum sample size is under these assumptions estimated to be 80 patients, that is, 40 per randomized group [29].

### Sample size interim analysis

Given the invasive character of IMBI and the burden for the patients, an interim analysis is pre-planned to test whether we can stop the study preliminarily assuming that the experimental group has a higher success rate than the control group and to monitor planned sample size assumptions. The sample size calculation for the interim analysis is based on the assumption that a success rate of 20 % in the placebo group is probably too high. Therefore, we assume a success rate of 10 % at 6 months in the placebo group, instead of 20 % success. If we detect a difference in success rate of 40 % between the two groups, the sample size is estimated to be 50 patients, that is, 25 per randomized group with a power of 90 % and a level of statistical significance of 2.5 % (one-sided).

### Statistical analysis

Differences in success rate between the groups will be tested with a Chi square test (univariate). Success is defined as at least 30 % pain reduction at 6 months. To account for cluster effects, a linear multivariate mixed model will be used to determine differences in pain score changes over time. A logistic regression model will explore possible predictors for success or failure of the procedure. The changes after 6 months of the outcome variables of the two groups will be compared with a Student’s t-test.

A trial-based economic evaluation will be performed, based on empirical data obtained in the RCT. The base-case economic evaluation will be performed from the societal perspective, including costs inside and outside the healthcare sector and will follow the, in 2012 published, international guidelines [30]. The time horizon of the economic evaluation will be 6 months. The intervention offered in this study is primarily expected to affect morbidity. So, health-related quality of life is considered as an important outcome in these patients. Therefore, a cost-utility analysis will be performed, with the number of quality adjusted life years (QALYs) as the primary outcome measure, based on the EQ-5D. Additionally, a cost-effectiveness analysis from a healthcare perspective will be performed in which pain reduction is used as measure of effectiveness. So, incremental cost-effectiveness ratios will be expressed as 1) the incremental costs per QALY (societal perspective) and the 2) incremental cost per additional patient with a clinically relevant pain reduction (healthcare perspective). The cost analysis will be performed according to Dutch guidelines for cost calculations [30]. Hospital resources use such as outpatient visits and diagnostic procedures will be registered by Case Report Forms. Study-related costs will not be included in the cost-analysis. For costs outside the health care sector, such as visits to the GP, physiotherapist, productivity losses, and out-of pocket costs, patients will be asked to fill out cost questionnaires with a recall period of 3 months at three occasions: at baseline, at 3 months and at the 6-month follow-up. Costs will be calculated by multiplying resources use with standard unit prices [30]. Standard sensitivity analyses (for example, using SF-36 for QALY calculation instead of EQ-5D) and bootstrap analysis will be performed to investigate the uncertainty surrounding the cost-effectiveness ratios. Based on the bootstrap results, cost-effectiveness acceptability curves will be constructed, showing for a wide range of cost-effectiveness threshold values, the probability that IMBI is potential cost-effective. The economic evaluation will only provide insight into the potential cost-effectiveness of IMBI, as IMBI is compared with placebo instead of a natural comparison (CAU). In a secondary analysis, information from the baseline cost questionnaires (covering a period of three months before entering the study), baseline pain scores and baseline quality of life, will also be used as an estimate of costs and effects of CAU.

### Monitoring

#### Data Monitoring Committee (DMC)

The major task of the DMC is safety monitoring. Any serious adverse event or suspected unexpected serious adverse reaction, which might be life threatening or liable to produce disability or deformity, is an indication to set up a DMC meeting. Another important task for the DMC is monitoring the efficacy for futility and monitoring the planned sample size assumptions, whether criteria for early stopping are met. The statistician of the DMC will be the only one during the trial who has access to the un-blinded data. He performs the (interim) analyses. Members of the DMC are (1) head of the department of orthopedic surgery and (2) statistician and internal medical specialist of Maastricht University Medical Centre (MUMC) the Netherlands. Each member has no conflicts of interest with the study or the study members. The advice(s) of the DMC will be notified upon receipt by the research committee to the METC that approved the protocol. With this notification a statement will be included indicating whether the advice will be followed.

This RCT will be audited and monitored by the Clinical Trial Centre Maastricht (CTCM); this organization is independent from the investigators and sponsor.

## Discussion

Methylene blue (10 mg methylthioninium) is an off-label medicine, usually used as a dye for therapeutic and diagnostic applications. To establish presumed efficacy and cost-effectiveness of IMBI for discogenic LBP, a grant was obtained from “The Netherlands Organisation for Health Research and Development” (ZonMw).

Preceding this RCT, a pilot study with 15 discogenic LBP patients was performed, to acquire information about efficacy, safety, feasibility, and acceptability of this intervention [31]. Information from this pilot study was used for designing this randomized controlled study. The RCT performed by Peng et al. was highly successful (89 %). Our pilot study indicated that IMBI was successful in 40 % of patients (at least 30 % pain reduction at 6 months) and this information was used to adjust the sample size calculation. Furthermore, the pilot study demonstrated that it was feasible to recruit a carefully selected population of patients with discogenic LBP and provided information about how to avoid “lost to follow-up.” Finally, the pilot study was used to provide information to the research funder and, consequently, enabled a large-scale RCT.

The use of invasive placebos for pain treatment interventions is currently under debate and questions are raised about whether it is justified to expose control group patients to risks of serious harm [32, 33]. Since 1955, the placebo phenomenon has been considered to have considerable clinically important effects [34]. This view was challenged when in 2001 and 2004 systematic reviews of clinical trials concluded that there was no evidence for placebo treatments having clinically important effects, except perhaps in patient-reported outcomes for pain [35, 36]. The authors later published a Cochrane review with similar conclusions [37]. Because in our study, the treatment’s main objective is pain reduction as experienced by the patients, we decided to use a placebo treatment as control. A placebo intervention, a mixture of saline, lidocaine, and contrast will be injected to establish if there is a pain relief surplus when adding methylene blue into the mixture instead of saline.

It is debatable if the use of the term ‘placebo’ treatment is correct for lidocaine shall be injected in the control group as well as in the treatment group. Local anesthetics are originally known for their inhibitory function on sodium channels, but they also have important anti-inflammatory effects [38, 39]. However, the former RCT [18], which was highly successful, also used intradiscal lidocaine for both study arms, and they found no large pain-relieving effect in the control group.

Providing evidence for cost-effectiveness of low back pain interventions is a stipulated condition from the Ministry of Health in the Netherlands. Without this evidence, the Dutch Public Health Insurance System will not reimburse low back pain interventions. Overall, there is lack of cost-effectiveness studies for interventional pain medicine; therefore, an important part of this study concerns a cost-effectiveness assessment.

This study will generate information for health care providers, patients, and health insurance companies, and results can be used in future guidelines and clinical practice algorithms.

Intradiscal methylene blue injection could potentially be a new treatment in specialized interventional pain centers for patients with discogenic LBP who do not respond favorably to conservative treatments. For these intractable discogenic low back pain suffering patients, there is currently no recommended pain treatment available; therefore, this intervention could become a “pain treatment of last resort.”

## Trial status

This is the protocol of an ongoing trial; patient recruitment is not completed at the time of submission.

## Abbreviations

BMI: body mass index (kg/m^2^)
CAU: care as usual
CDS: controlled disc stimulation
CTCM: clinical trial centre Maastricht
DMC: Data Monitoring Committee
EudraCT: European Union Drug Regulating Authorities Clinical Trials
IMBI: intradiscal methylene blue injection
LBP: low back pain
MUMC: Maastricht University Medical Centre
MB: methylene blue
RCT: randomized controlled trial
NRS: Numeric Rating Scale
MRI: magnetic resonance imaging
PGIC: Patients Global Impression of Change
PSI: pound per square inch
QALYs: quality adjusted life years
QOL: quality of life
ZonMw: The Netherlands Organisation for Health Research and Development
